# How to communicate climate change ‘impact and solutions’ to vulnerable population of Indian Sundarbans? From theory to practice

**DOI:** 10.1186/s40064-016-2816-y

**Published:** 2016-07-29

**Authors:** Abhiroop Chowdhury, Subodh Kumar Maiti, Santanu Bhattacharyya

**Affiliations:** 1 Department of Environmental Science and Engineering, Indian School of Mines, Dhanbad, 826004 India; 2 Tagore Society for Rural Development, 46B ArabindaSarani, Kolkata, 700005 India

**Keywords:** Social work, Climate change adaptation strategies, Community mobilization, Wealth rank tool, Micro-finance institution (MFI), Communication, Alternative income generation activity, Disaster management, Non-Governmental Organization (NGO), Mangrove conservation, AILA, Endemic knowledge

## Abstract

**Introduction:**

Global consciousness on climate change problems and adaptation revolves around the disparity of information sharing and communication gap between theoretical scientific knowledge at academic end and practical implications of these at the vulnerable populations’ end. Coastal communities facing socio-economic stress, like densely populated Sundarbans, are the most affected part of the world, exposed to climate change problems and uncertainties. This article explores the successes of a socio-environmental project implemented at Indian Sundarbans targeted towards economic improvement and aims at communicating environmental conservation through organized community participation.

**Case description:**

Participatory rural appraisal (PRA) and the wealth rank tool (WRT) were used to form a “group based organization” with 2100 vulnerable families to give them knowledge about capacity building, disaster management, resource conservation and sustainable agriculture practices. Training was conducted with the selected group members on resource conservation, institution building, alternative income generation activities (AIGA) like, Poultry, Small business, Tricycle van, Organic farming and disaster management in a participatory mode. The climate change ‘problems–solutions’ were communicated to this socio-economically marginalized and ostracized community through participatory educational theater (PET).

**Discussion and evaluation:**

WRT revealed that 45 % of the population was under economic stress. Out of 2100 beneficiaries’, 1015 beneficiaries’ started organic farming, 133 beneficiaries’ adopted poultry instead of resource exploitive livelihood and 71 beneficiaries’ engaged themselves with small business, which was the success stories of this project. To mitigate disaster, 10-committees were formed and the endemic knowledge about climate change was recorded by participatory method validated through survey by structured questionnaire. As a part of this project 87 ha of naked deforested mudflat was reclaimed with endangered mangroves involving target community members aimed to sequester CO_2_, control soil erosion and act as a barrier during natural disasters.

**Conclusion:**

This case study concluded that participatory method of communication, aiming not only to communicate theoretical knowledge, but also to devise adaptation strategies through conservation of endemic knowledge, popularizing sustainability through Micro Finance Institutions and promoting AIGA along with motivating vulnerable community to restore degraded forest lands, could be a effective solution to practically combat climate change problems.

**Electronic supplementary material:**

The online version of this article (doi:10.1186/s40064-016-2816-y) contains supplementary material, which is available to authorized users.

## Background

The problems of climate change, mainly sea level rise, global warming, increasing instances of disasters, changes in agro-production have a profound effect on the coastal communities. The deleterious effect of climate change on these ecosystems, environment and their productivity gives rise to complex human impacts affecting food security, lower coping capability with ever increasing natural disasters and fresh water shortage crises (Kilroy 2015).

Early communications on climate change is mostly limited to scientific findings and synthesis of reports (Moser 2010). But this information originating at the researchers/scientist/environmentalist’s end should percolate to the resident populations and properly dealt with the managers (Tribbia and Moser 2008). Closing the gap between science and practice is a pressing ordeal bridged by the managers who regulate this flow of information from source to users (Cash et al. 2003). Another constraint in communicating climate change is the less impetus given to the human behavioral components in policy formulation and more weightage are given to the scientific fact-findings and technological aspects. Unsustainable ‘quick–win’ mindset of the people is mostly responsible for resource exploitive and non-green practices (Spence and Pidgeon 2010). So educating the local population is the foremost duty of an environmental manager to communicate climate change issues. Environmental threats can be mitigated with the dynamic participation of the local community and this ‘bottom up’ (from lowest strata of society to the policy making officials) mode of management is the effective process to battle this world threatening climate problems and communicating solutions (Carvalho et al. 2012). Community based approaches (CBA) target participant empowerment and a structured mechanism to communicate ideas from bottom to top (Allen 2006). Researches show that CBA is socio-economically effective tool in forest conservation and give positive outcomes (Chen et al. 2013; Robert and Rebecca 2006). Growth of these CBA’s in environmental problems is due to its cost–effectiveness and preference of donors to this ‘community component’ for easy penetrability of the information and ideas to the lowest strata of the society. Communication involves a large number of discrete processes like framing messages through pictures and articulation, through the tale of life experiences for presenting an issue or event (Chong and Druckman 2007; Spence and Pidgeon 2010). Thus the participatory style of awareness campaigns is effective in imparting consciousness about negative impacts of climate change. Social learning has many benefits than individual communication. It results in increased in shared knowledge, trust between members and induce democratic decision-making power to community members (Biedenweg and Monroe 2013).

Coastal region comprises of only 4 % of the world’s land area, but one-third of the world’s population resides there (Cochard et al. 2008; UNEP-WCMC 2006). Deltas are the world’s most vulnerable yet most populated regions with respect to the climate change influences. About 300 million people inhabiting the major deltas of the world (populaion density: 500 people/km^2^), are at an escalated risk due to climate change causing sea level rise and increase in natural disasters along the coast (Ericson et al. 2006; Nicholls et al. 2007; Chowdhury et al. 2016).

The Ganga–Brahmaputra–Meghna delta has already been labelled as the ‘Extremely vulnerable’ zone in the terms of population displacement due to climate change and raising ocean levels by 2050 (Ericson et al. 2006; Nicholls et al. 2007; Chowdhury and Maiti 2014; Chowdhury et al. 2016). It covers an approximate area of 10,000 km^2^, with one of the highest population density in the globe, shared by two nations, India (40 %) and Bangladesh (60 %) and is the abode of the world’s largest contiguous mangrove forest of Sundarbans (Maiti and Chowdhury 2013). In an estimate it is argued that mangroves are disappearing at an alarming rate of 2 % per year which is higher than any other endangered ecosystems, like coral reefs or tropical rain forest, due to anthropogenic interventions like habitat modification for agriculture and aquaculture along with pollution problems (Dasgupta and Shaw 2013; Chowdhury and Maiti 2016a). This austere amphibious terrain infested with malaria causing mosquito, snakes, crocodile, tigers and regularly battered by cyclones and storm, is populated by ostracized section of society (reserve castes). Social organization of the Indian part of Sundarbans is discussed in details in Additional file 1. Low education levels, chronic health problems, excess labor migration, a low skill base, lack of access to official credit, low savings, under employment, high dependence on government and poor infrastructure (housing, roads, embankments, ponds, waterways, marketing, electricity and communication) are indicators of a poor livelihood asset base in the region. And this compels the inhabitants to engage in resource exploitive activities like poaching and deforestation.

**Fig. 1:**
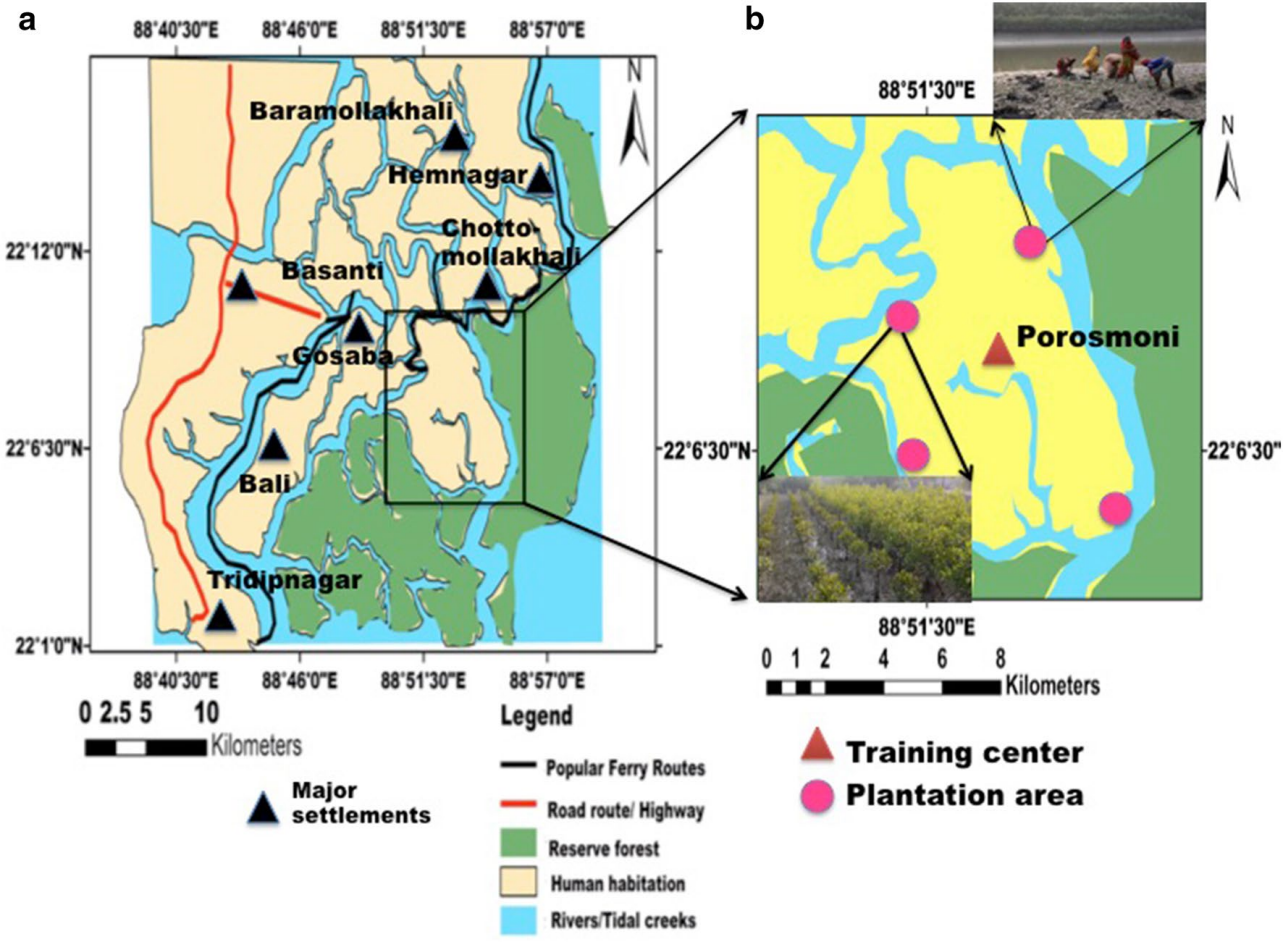
**a** North-eastern part of Indian Sundarbans, **b** the project area of Satjelia is zoomed out for better visualization with training center at Porosmon and the plantation areas

 Researchers opined that adaptation to climate change required to build the resilience especially for the economically compromised section of society residing in the most vulnerable part of the world (Costello et al. 2009; De Souza et al. 2015). Conscious participation of the resident human population is the only way to communicate climate change issues and conserving mangroves in this sensitive region as a solution to these problems. For example, Satjelia Island (Fig. 1) (Area: 225 km^2^) is the last habitable island of Indian Sundarbans communicable only by waterways, with sporadic electricity supplywhere the majority of population falls under category of Bellow Poverty Level (BPL) and belonging to ostracized minority population (SC/ST, Muslim), where this participatory mode of communication method has been applied (Additional file 1: Table S1).

The process and outcome of the communication of environmental degradation to grass-root level population by understanding and linking livelihood, trainings, mangrove plantation programs and vulnerability is the focus of this paper. Projects can gain sustainability by the presence of strong local institution and community participation. The organogram and concept of initiation of this project is discussed in details in the Additional file 1: Fig. S1. Women savings groups were experimented in various parts of the country since 1980’s, in which these Self Help Groups (SHG’s) were funded by monetary inputs from different funding agencies, both governmental and foreign, and local NGO’s act as a distribution center for disbursement of such credits (Premchander 2003). Group members and the charitable organizations effectively manage micro-finance institutions (MFI) system and group participation plays a pivotal role in its success and sustainability (Benson et al. 2001). Local NGO’s activities play a primary role in managing post disaster crisis in different parts of the globe (Benson et al. 2001). Here we have targeted 2100 marginalized families of the Satjelia island to communicate climate change affect and solutions through training programs and theatrical performances by organizing them in a group based organization with positive outcomes. This could act as an effective way to communicate these problems to the grass-root level and narrow the gap between the affected population and educated academia.

## Methods

### Procedure for selecting group members

Participatory rural appraisal (PRA) has been used to select group members in accordance with the community after a baseline survey conducted randomly on the resident population of Satjelia Island prior to the formulation of the project (Bar-On and Prinsen 1999; Chambers 1994). Wealth ranking tool (WRT) was used to classify the vulnerable population into wealth classes that is elucidated in Table 1. WRT is a popular and widely used socio-economic tool that can assess the economic status of a target area, but the indicators differes from place to place, as every region have their own socio-economic complexicites that need to be considered while formulating the categories/criterions for this tool (Scoones 1995). In wealth ranking program four categories were made, i.e. A-Ultra poor, B-Poor, C-Less poor and D-others. Groups were structured with the people of “A” and “B” category only. Those individuals who fall under the “C” and “D” category were not considered for the membership in the groups. The classification was set according to the result of the wealth rank. Ranking by WRT has been done through communication with the local populace. No set rules are there and compliance to any one criterion is enough for admission of a family of these categories (either A or B). The local population of the village communicated the criterion for wealth ranking in accordance to the participatory process during the wealth ranking procedure. For example, the Wealth category ‘A’ has the criterion of ‘widow’. A widow of a senior school teacher of any governmental aided institution gets a pension amount, so only being a widow does not ensure the admission of that individual/family into the category as she is not under any socioeconomic stress and this may be a consideration of the villagers for not placing her in the ‘A’ category. Similarly, in the case of ‘physically not fit to work’ criterion, if that person has an alternative income source then he/she would not be qualified to be admitted to the category ‘A’.

**Table 1:** Wealth-rank classification criterion

Sl. no.	Wealth category	Criteria
1	A (ultra poor)	1. Land less 2. Widow 3. No scope for rearing live stock 4. No agricultural land 5. Work as daily labor 6. Divorcee women 7. Solitary women 8. Capture fingerling in river for feeding their family 9. Only one earning member in family 10. Work as agriculture labor 11. Small hut for living 12. Reside at road side 13. Physically not fit to work 14. Reside at river side 15. Only having land for residing
2	B (poor)	1. Small land 2. Less income 3. More than 4 members in a family but income less than 1000 INR/month 4. Small land for cultivation
3	C (less poor)	1. At least 2 earning members in the family 2. Having scope for rearing live stock 3. At least 1-acre land 4. Sufficient land for cultivation 5. Family member migrated in other states for earning
4	D (others)	1. Service men 2. Pension holder 3. Businessmen 4. Large agricultural land 5. Well built house for residence

**Fig. 2:**
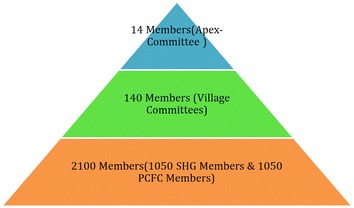
The organizational pyramid (2100 members)

The selected members in category ‘A’ of Wealth rank, is again asked for their willingness to join the group. Using WRT 2100 beneficiaries’ (Households) were selected. Each household has one representative in the group. A total of 140 groups were organized of which 70 were female membered SHG and 70 male membered Primary Committee for Forest Conservation (PCFC) groups. The organization is explained in Fig. 2 and Additional file 1: Table S2. From each hamlet (7 hamlets in total) one ‘Village Committee (VC)’ was selected having equal representatives from SHG and PCFC groups. Each hamlet has 20 groups (10 SHG’s and 10 PCFC’s). Above the VC one ‘Apex Committee (AC)’ was selected from the 7 VC. Over the different organizational hierarchy, group members play the sole role in selection of representatives in VC and VC members further select their AC members in a democratic framework. The AC is the topmost tier in the organization having the maximum role in decision-making process.

The administration protocol of the organizational framework is totally democratic. Each group (SHG and PCFC) has one President, one Treasurer and one Secretary who were democratically elected by the group members. VC which has 3 representative members from each group, and hence each VC has 20 members one from each PCFC’s and SHG’s in a hamlet. Each group has a savings account managed by the treasurer and supervised by the group leader and training coordinator. All members have to deposit INR 10/Month or 0.15 US Dollar/Month (minimum) in the account, which act as a disaster management fund to be used during a crisis. Members can apply for lone to start sustainable alternative livelihood or other activities. The plea/application for lone need to be passed through the group hierarchy and only after that the applicant can be given the stipulated loan and loan recovery would be done by the group members. Two members from each VC represent the AC so there is only one AC in Satjelia island with 14 members. This endeavors target in introducing effective MFI’s to maintain the sustainability of the environmental friendly program components.

### Collection of local contribution (LC)

According to the project agreement with donor 6.9 % LC must be collected from beneficiaries and local coordinating NGO. But raising 6.9 % LC from the beneficiaries’ is an arduous and seemingly unethical task, as they don’t have the financial position to pay. So to make it simple, man-hours of attendance in the training and their contribution in mangrove plantation and conservation have been converted into monetary contribution of the group according to the current labor rates in the region and treated as LC. This has a two fold benefit, (1) the required money for LC has been raised, and (2) the attendees of the program, i.e. the beneficiaries’ understand their contribution and treat the trainings sessions as a program where they have also monetarily invested and not a mere philanthropy of foreign donors, which in turn improved their participation.

### Training programs

Trainings and awareness campaigns were conducted on various aspects of climate change issues and capacity building programs. These programs were (1) Training on sustainable agriculture, (2) Poultry, (3) Organization building training, (4) Disaster Management, (5) Climate change issues, (6) Participatory educational theater (Chamberlain et al. 1995), and (7) Resource conservation.

Collaboration with multiple stakeholders was encouraged in these training programs. Governmental bodies like MOEF and CC (Ministry of Environment, Forest and Climate Change, Government of India), Agricultural Department (Government of West Bengal, India), Veterinary Department (Government of West Bengal, India) and Disaster Management Authority (Government of West Bengal, India) helped in these programs by providing expertise and resource person. Trainers are previously briefed about the socioeconomic and cultural condition of the community before the training to deter any avoidable complications during the session and easy accessibility of the information/skills from resource person in the target population. Trainings generally range between 2 and 4 days depending on the responses’ of the trainees. And no individual is allowed to attend two trainings expect for the same type of trainings like poultry or resource conservation. The details on training content have been discussed in Table 2. For example an individual receiving training on sustainable agriculture would not be allowed to appear for training in poultry.

**Table 2:** The training details and courses covered

Name of the course	Who will undergo	Tentative course contents	Purpose
People’s participatory plan and monitoring	Members of Hamlet Committee (PCFC and SHG)	1. Development understanding from various schools of thought, different development concept, methods and tools. Understanding participatory rural appraisal (PRA), rapid rural appraisal (RRA) etc. 2. Participatory process: different approach and practice 3. Identification of problem, analysis, Decision making, planning and monitoring through participatory process	1. Enabling the people so that they can identify, analyze and realize their problem towards community convergence action
People’s institution building and management	Members of the APEX committee, Leaders of SHGs and Members of FCC Federation	1. Character of people’s institution 2. Planning from aim to accomplishment 3. Organizational dynamics and managing organizational conflict 4. Span of control and functional authority 5. Responsibility and accountability 6. Strengthening motivation 7. Leadership and change—out put and intervening variables	1. Capacity building of the members so that they can build, hold and run their own organization
Resource conservation. Pollution and environment management	Members of PCFCs	1. Environmental complex—dynamic nature of environment 2. Ecosystem dynamics—plant and its environment 3. Impact of man on environment-applied ecology of individual, population and ecosystem 4. Distribution of natural resources. Renewable and non-renewable factors 5. Trend of exploitation of natural resources 6. Trend of pollution of environment 7. Plant responses to pollution 8. Mangrove ecosystem—distribution of littoral fauna—animal societies and territoriality 9. Techniques of controlling environmental pollution including biological controls 10. Social forestry and mangrove vegetation 11. Participatory forest management	1. Understanding and realization regarding the importance of geo-specific environmental uniqueness of Sundarbans and its management
Sustainable agriculture and vermicompost	Identified farmer from target community who have ownership of agricultural land	1. Agro forestry and nursery management 2. Cultivation potential herbals 3. Soil testing and integrated nutrient management 4. Watershed management 5. Bio fertilizer, Organic manure, vermin compost	1. Enablement towards agricultural sustainability
Small business	Identified those who totally depended upon Forest for their livelihood and does not have own land	1. Identifying the trade 2. Planning and execution 3. Business capital 4. Pricing 5. Marketing	1. Capacity building of the small business man so that they can develop their own business—alternative income generation activity (AIGA)
Disaster management	Families who lives in disaster prone area	1. Identification of deeper and weaker sections of the community 2. How to minimize risks 3. Preparation before disaster 4. Security 5. Risk analysis 6. Preparation of risk maps 7. Evacuation plans	

### Participatory educational theater (PET)

Chamberlain et al. (1995) first used PET in AIDS awareness program amongst the affected population in Africa. In this project this method of communication has been modified as per local demands. ‘*Yatra*’ or street theaters are popular mode of entertainment for the local populace, which is conducted by mobile troupes. Here participants/volunteers have been selected from the target population belonging to the category ‘A’ and ‘B’, and trained to perform these ‘shows’ to convey the messages of disaster management and sustainable livelihood in the form of musical stories. These theaters are performed in religious ceremonies and during market day to attract most crowds. As during market day many people come from far-flung islands of delta, these messages are communicated to them. The success of sustainable agriculture and alternative livelihood is also communicated through these ‘shows’.

### Plantation program

**Fig. 3:**
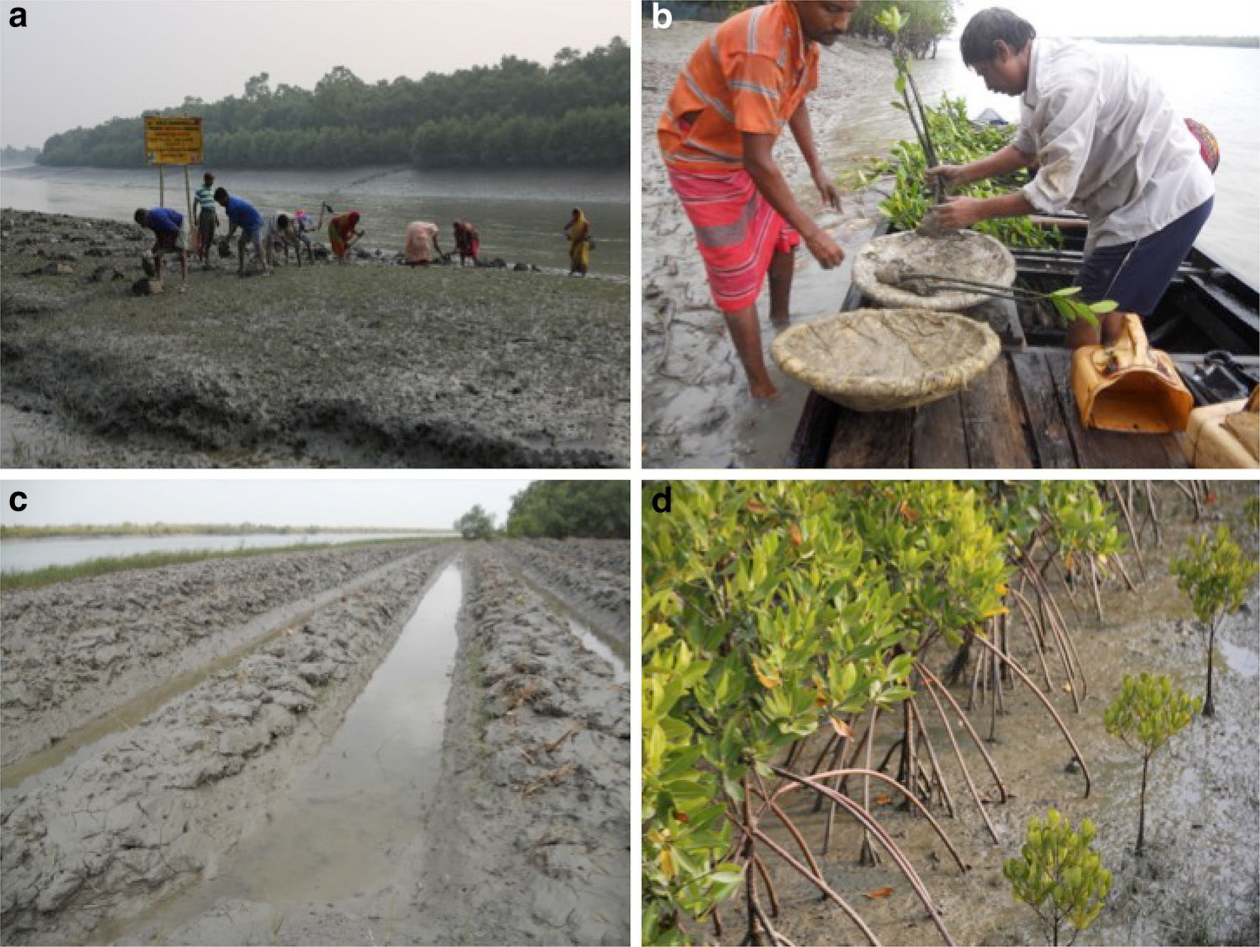
**a** Clearance of *Porteresia* sp. patch and digging of pits for plantation of mangroves, **b** transportation of saplings to plantation area via boat from nurseries, **c** trench digging for *Rhizophora* sp. plantation, **d** the *Rhizophora* sp. plantation with prominent stilt root

**Fig. 4:**
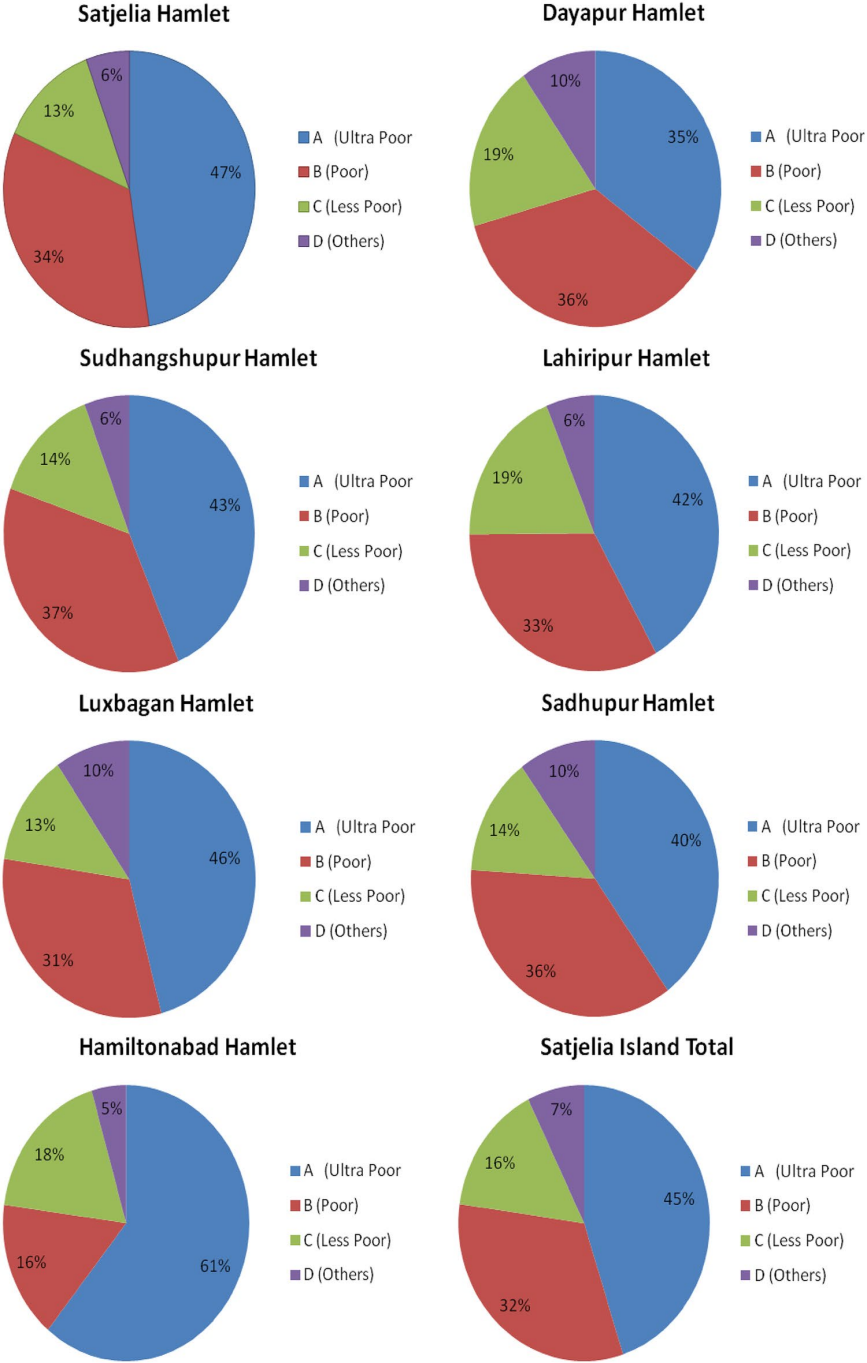
Result of the wealth rank classification in seven hamlets (Satjelia, Dayapur, Sudhangshupur, Lahiripur, Luxbagan, Sadhupur and Hamiltonabad) and the total Satjelia Island. Household covered during the WRT survey out of total recorded households (Census 2011), in each hamlet are as follows; Satjelia- 71 %, Dayapur- 64 %, Sudhangshupur- 78 %, Lahiripur- 59 %, Luxbagan- 79 %, Sadhupur- 77 % and Hamiltonabad- 96 %

Mangrove species produce propagules that are relatively easy to collect and plant. Propagules are directly planted particularly for *Rhizophora* spp., but for other mangroves, seedlings and saplings have grown to a height of 0.3–1.2 m beforehand at nursery. Replanting mangroves is a useful first step but to restore the ecosystem, to protect the embankments by process of soil consolidation. The process of plantation follows this order (1) clearing of *Porteresia* sp. patch to plant new mangrove saplings, as it hinder the growth of other mangrove plants by covering the sediments by thick mat of fibrous root system and competing with saplings of other halophytic species for nutrients (Fig. 3a), (2) saplings are transported in wooden boat/dinghies to plantation sites from the nursery (Fig. 3b), (3) digging of pits/trenches for plantation of saplings (Fig. 3c), (4) excavation of trenches for plantation of *Rhizophora* spp that possess distinct stilt roots (Fig. 3d).

## Result and discussion

### Outcome of the wealth rank classification

For wealth rank classification 7195 households were selected for the survey out of 9800 total households in the Satjelia Island (including all the 7 hamlets). First the program details and aim was communicated to most of the households by the program employees. In this process villagers were gathered in the center of the village and told to identify the categories, set classification criterion and identify the probable members for the program. This has also given a picture of the socioeconomic, demographic composition of the last habitable island of Indian Sundarbans and the results are explained in Fig. 4. It is evident that according to the respondents, 43 % of the total population of this island are in Category ‘A’. Hamiltonabad hamlet has the highest percentage of “Ultra poor” population (61 %) whereas Dayapur hamlet has the lowest (35 %) amongst the 7 hamlets.

### Outcomes of training program

**Fig. 5:**
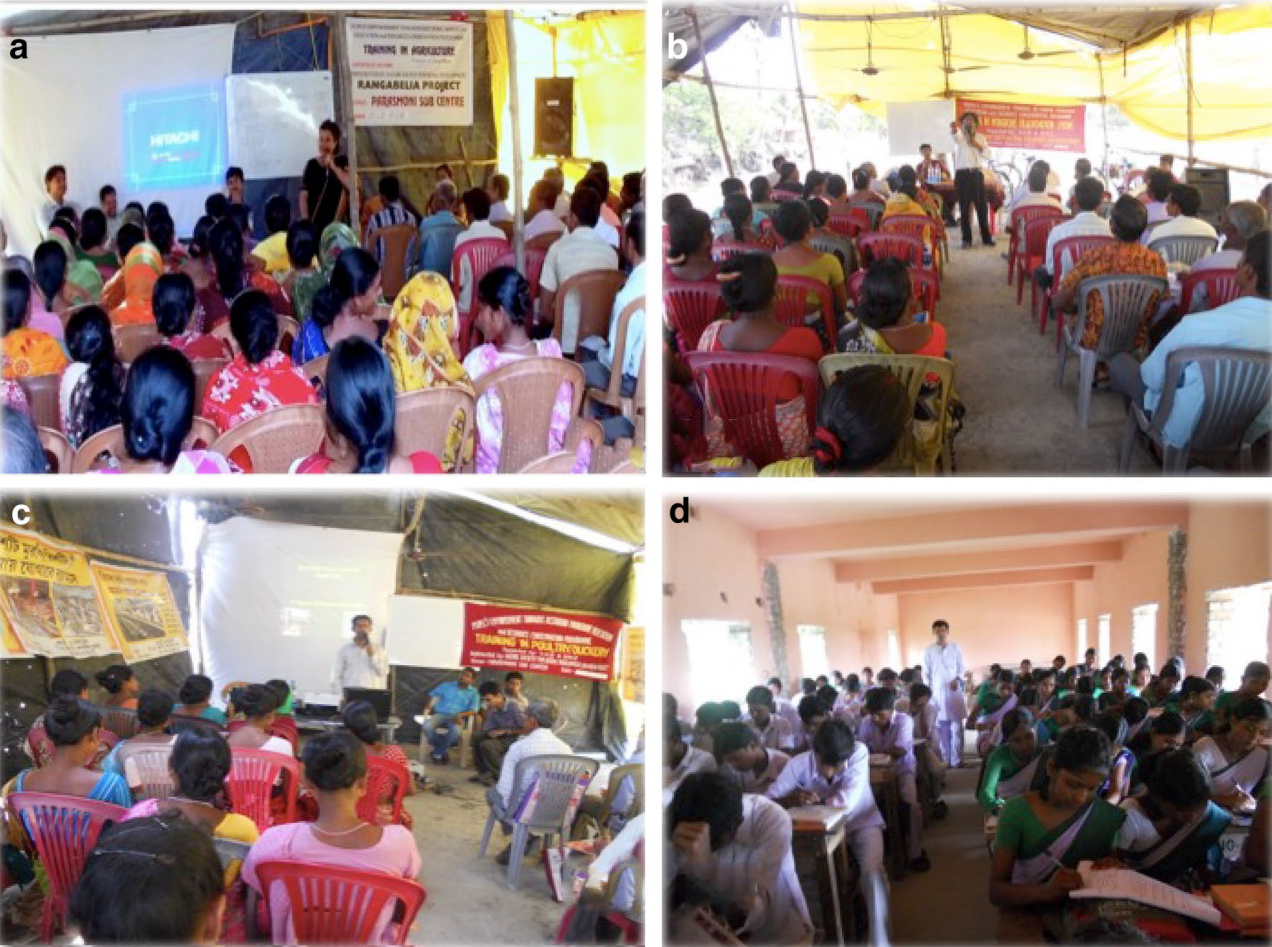
**a** Training in agriculture organized at porosmoni, **b** PIBM training to build organizational skills of the group members, **c** poultry training as an alternative source of livelihood (AIGA), **d** quiz contest at local school on climate change issues and to promote environmental awareness

Trainings were conducted on various aspects of climate change and climate change mitigation through 2012–2015. The batch size is kept small and managable for better communication between resource person and trainees. The group-based organizations were responsible for selection of beneficiaries’ who would take part in these trainings according to their need and interest. The trainings enlist active participation of the trainees. Breakfast and lunch were provided. Figure 5 showed different trainings conducted with group beneficiaries’.

Training content encompasses a wide selection of topics from development of organization, promotion of sustainable agriculture, disaster management through the creation of the disaster management team, promotion of alternative livelihood to deter unsustainable resource extraction that is responsible for the deforestation of the endangered mangrove. The details about the training contents and objectives has been elucidated in Table 2. The resources given to the attendees were in the form of booklets, information and education pertaining to the particular scope of the program component. In the closing session of Alternative Income Generation Activities (AIGA) such as small business training, poultry and sustainable agriculture resources has been given that would help the trainees to go their own AIGA. For example, Feeder, drinker, 40 birds and 1 month worth of feed was given to each trainee to start their own poultry. Likewise, after successful implementation of the training, organic fertilizer/pesticides (Neem oil, vermicompost etc.) has been distributed amongst the trainees to start their own organic farming initiatives. Monetary support was approved by the Apex committee and funded by the donor and implementing NGO, to be reimbursed to the candidates who have bought Tricycle van to augment their income. In the islands, Tricycle vans are used to carry passengers in exchange of money, so this substancially augmented the income of the candidates engaging in the trade. Same is the case for the grocery shop enterprenuers who have received monetary support from the funding agency and implementing NGO to start their business.

The main topic for the ‘Resource conservation’ training focused on what are resources, identifying different resource and their uses. Different natural resources of the Sunderbans area were discussed with special focus on the ethnobotany of the mangrove plants and their uses. Effect of climate change in alteration of biodiversity was the pivotal point in this discussion and inputs from participants have been encouraged. Interactive sessions were conducted where the beneficiaries’ of different age group participated to communicate the changes in biodiversity that they have noticed since 1980’s. Mature group members (35–45 years old) have articulated that previously fishes were abundant in the creeks but over the years the quality and quality of the rare edible carps like Hilsa (*Tenualosa ilisha*), Bata (*Labeo bata*) have decreased. Hilsa (*T. ilisha*) was in high abundance during monsoon season till 1995 but increased cyclonic disturbances the catch of that particular fish have had dwindled in the region ever since.

**Fig. 6:**
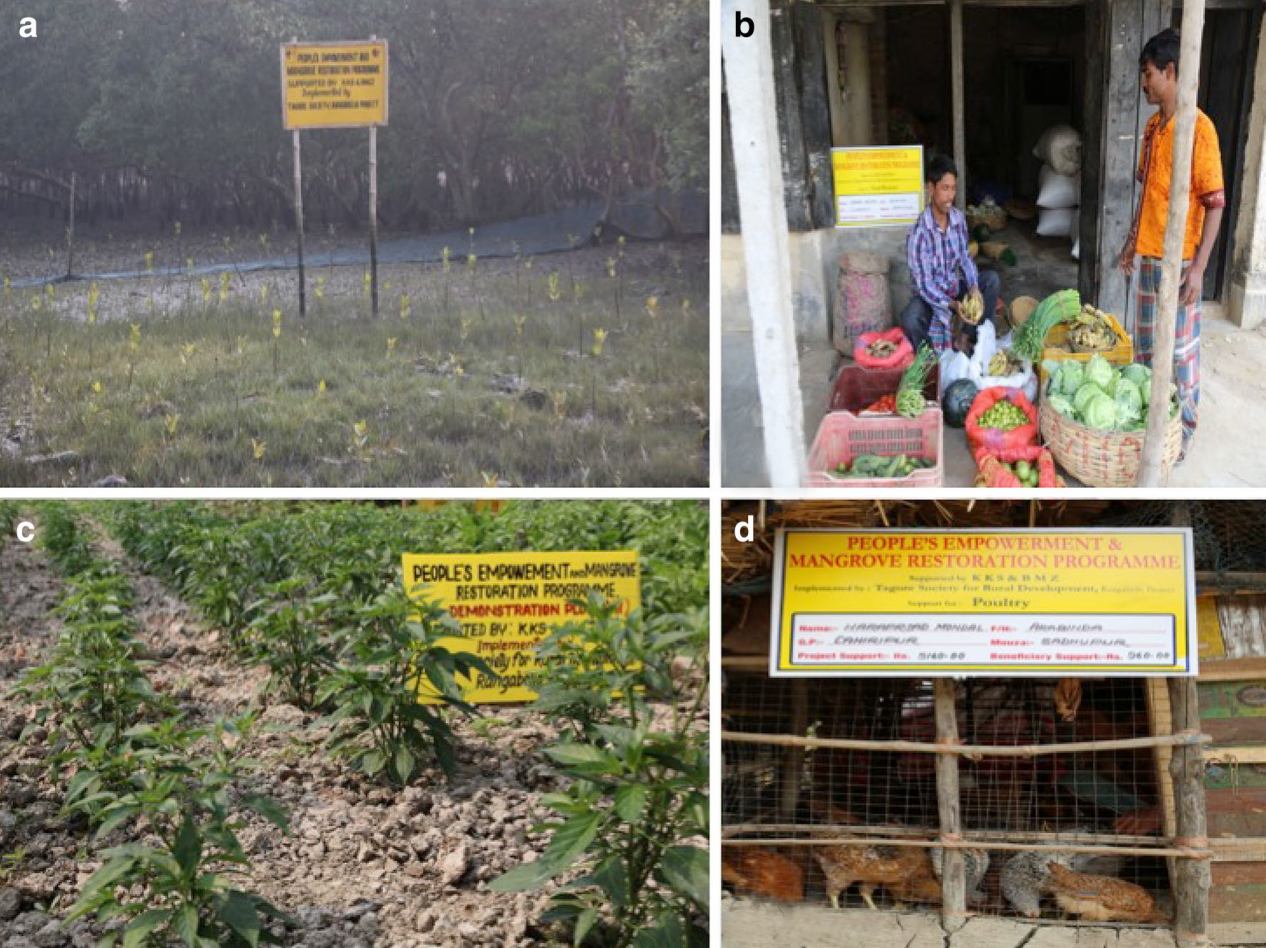
The initiatives taken by the group members; **a** reforestation of naked mudflat with mangroves for resource conservation, **b** grocery shop as an sustainable initiative for alternative resource friendly livelihood, **c** chilli, tomato, eggplant garden with organic agriculture, **d** poultry initiative as a sustainable resource friendly mode of alternative livelihood

Plantation program was one of the integral parts of this project that directs towards a sustainable solution towards climate change adaptation. AIGA activities were aimed towards sustainable livelihoods so that the beneficiaries’ do not indulge into nature exploitive sources of income like fishing tiger prawn, logging of mangrove trees, limited use of chemical fertilizer in agriculture (Maiti and Chowdhury 2013; Chowdhury and Maiti 2016b). This in turn would help in preserving/conserving this fragile mangrove habitat. Settlement in these islands large tracks of mangrove forests have been cleared, which need to be re-vegetated for the dual purpose of mangrove conservation and protecting the embankments during any natural calamities like cyclone/storm surges (Chowdhury and Maiti 2014; Chowdhury et al. 2016). The PCFC members actively took part in the plantation plan. The PCFC members also monitored protections of mangroves at Satjelia islands (Fig. 6a). Meetings with local Eco-development Committee (EDC) were also organized. Between 2012 and 2013 sixteen number of ‘*Bana Raksha’* Committee or Forest Protection Committee meeting were conducted. The average attendance at these meetings was 15. These helped in enlisting community involvement in the plantation activities. Local Panchayet members also attend these meetings and gave their full cooperation to the project for implementing activity in their area.

### Training and implementation of sustainable alternative livelihood

Sustainable alternative livelihood suitable for the region was promoted through trainings. Foremost amongst them were poultry and duckery and other being grocery shop and cycle van (tricycle used to carry people in lieu of fee). In 2012 and in 2013 total 71 numbers of beneficiaries initiated small business with average profit of 1553.91 INR/month (23.16 US Dollar) as communicated by the beneficiaries’ and they have recorded in their profit/loss book. 249 members applied and started transportation enterprenuership through ‘Tricycle van’ and they make an average profit of INR 1800/month (26 US Dollar). AIGA activities like grocery shop (Fig. 6b) were practiced by the group members and that augment their income making these trade popular amongst the practitioners. This was a safe alternative than to venturing deep into conserved forest with legal and life risks to catch fish/prawn.

Sustainable methods of agriculture were encouraged. Use of herbal pesticide like neem oil and compost in place of chemical fertilizer was promoted to maintain the health of the agricultural fields. Composting techniques were demonstrated and farmers were encouraged to be “model farmer” in the community to cultivate a part of their field in organic methods. This would motivate the other community members to follow suit. Between 2012 and 2013, 126 beneficiaries’ have committed to practice sustainable agriculture in part of their land. But after successful implementation of the program by the ‘pilot farmers’, more group members  also got interested to adopt organic farming methods. But none have applied the method in all of their farmland as they are uncertain of the outcome. So by participatory method it was decided that only a part of their land would be used for organic farming. The average size of their cultivated land is 0.32 acres and the crop mainly cultivated by beneficiaries’ were paddy, potato, pumpkin, ladies finger, cabbage, eggplant, radish, cauliflower etc. (Fig. 6c). 140 farmers agreed to create ‘Demonstration plot’ which could be shown to other community members (non-group) the success of the sustainable agriculture methods. The location of these plots were communicated to wider community during PET session, through meetings with Gram Panchayet (local governmental decision making body) and other stake-holders. As this component of Organic farming have a far reaching affect on the health of the agragerian soil and hence related to the socio-economic betterment of the populace, permission was availed from the funding agencies for the extra members applying for help in sustainable agriculture. Use of chemical fertilizers and pesticides have negative effect on the fertility of the land, making soil more dependent on those fertilizers for consecutive yields, but organic farming is a sustainable method of agriculture that improve the soil health.

### Poultry and rearing of birds

Training in poultry was promoted to rear birds as a sustainable profit-making livelihood. Between 2012 and 2015, 133 beneficiaries received support for poultry. Before starting poultry interested beneficiaries’ were advised to seek suggestions of Block Livestock Development Officer (BLDO). According to the expert suggestion, distribution of Road Island Red (RIR) breed and purchase of 21 days old chick in order to minimize the transportation hazards for chicks was followed. It was also confirmed from the BLDO that beneficiaries could get medicine free of cost from the Block Animal Health Unit. The poultry farms were successfully set up and non-group members were also taking interest in the process (Fig. 6d).

### Conservation of endangered mangroves

Scattered mudflats of 87.07 ha were brought under mangrove plantation in between 2012 and 2013. Approximately 140,843 saplings were planted (Satjelia Island) out of which 96,531 saplings survived by the end of 2015. The selected species for plantations were in endangered category as per IUCN red data book i.e. *Brugeria gymonorrhiza*, *Brugeria sexangula*, *Rhizophora mucranata*, *Avicenia* spp., *Ceriops decandra* and *Xylocarpus mekongenis* (IUCN 2014). 355 sacks (700 propagules/sack) of *Avicennia* spp. progules collected by local population that floats out from the reserve mangrove patches during high tide, were scattered in the mudflats during the plantation period. These *Avicennia* spp. that are tolerent to high salinity stress, have shown a profuse growth and a comparatively better survival rate (81.5 %) than the saplings (68.5 %). Previous works by Chowdhury et al. 2016, have also indicated that *Avicennia marina* can tolerate high salinity stress resulting due to the influx of salt water from sea during storm surges like AILA (2009). This trait makes one of the most suitable colonizers outcompeting the salt sensitive species, due to sea level raise and climate change. PCFC/SHG members were employed in this plantation process (Fig. 3) and given wages as per standard labor rates. Community members in supervision of the group members have taken part in this plantation program. The donor agencies funded the purchase of saplings and also the re-embursement of the labor rates charged by the plantation team.

Mangroves act as an effective carbon sink (Donato et al. 2011) and sequester highest amount of CO_2_ (than any other non-mangrove forest types) which approximately amounts to 100 tons of CO_2_ per hectare (ha) and also stabilize the soil particles to control erosion (Harty 1997). Hence, this plantation program was aimed to scale down the ominous effect of climate change as well as to form a natural barrier against any disaster. Mangroves have the unique ability to stabilize the soil particles, thus able to control the erosion. So, in future these plants can be effective to reduce the erosion of embankments of these areas. The huge root system of the mangrove flora has the ability to slow the tidal wave energy, this feature also helps to reduce the erosion. Then, in long term Mangrove would protect the country from both ill effect of climate modification and also from erosion of land. Works of Chowdhury et al. (2016) highlights that the climate change/sea level raise was responsible for the exclusion of true mangrove varieties (Rhizophoraceae members and plants with prominent physiological/phenological/morphological adaptations to combat salt stress) with salt tolerant/invasive species in central parts of Indian Sundarbans between 2008 and 2013. So sea level raise and climate change have deleterious influence on the mangrove ecology and re-vegetating the naked/deforested mudflats were a small step taken through this climate change adaptation project to conserve the mangrove varieties as well as increasing CO_2_ sequestration. And this plantation program was required to motivate the community members towards revegetating the deforested mud flats with mangrove plants and is an indispensable part of this project aimed at improving community level adaptation strategies to battle climate change issues in vulnerable tropical coasts.

### Training on disaster management and formation of committee

In disaster management trainings, special emphasis has been given to local knowledge of what the beneficiaries’ have faced during disaster and their view on how to adjust to these calamities. Participatory methods were employed where the discussion sessions were organized in each of the disaster management trainings. There the trainees were told to discuss about their decision making process during an impending natural disaster like cyclone/storm surges. That information is the traditional knowledge handed down through the generations of the trainees as most of them have been directly or indirectly related to fishing/aquaculture for livelihood and has to face storm/disasters in a regular basis and hence have adaptation skills to escape/survive during those disasters. And the project workers closely monitored these informal interviews and during subsequent group meetings those information were cross-checked with other group members. Again, this information was cross-checked with non-group members through snowball survey/random survey through easy designed questioner. The project workers and field staff conducted a total of 500 household surveys to validate this information. The structured questioner directly asked them whether they agree with the particular information about the disaster or not and only two responses were allowed, i.e. (1) ‘Yes’ and another (2) ‘No’. About 60 % of the information shared by the trainees found above 75 % acceptance amongst the group members and non-group members. A disaster management committee had been formed where the experienced members gave the young members’ education about the prediction of impending disaster. Only the information cross-checked in-group meetings and found consistent amongst the local population was communicated to the committee members. The beneficiaries’ have suffered during the cyclone and communicated their experiences in the training. They assisted each other during the crisis extending hand of friendship, sharing resources irrespective of caste and social positional differences. Same helping mentality between the sufferers was also seen during any flood related disaster. During the period of 2012–2013, 10-disaster management committee has been formed by the project. Each committee has 10 individuals trained to combat disaster problems The main points of training were-dangers from natural calamities and how to prepare for the disaster, uses of disaster fighting equipment, how to increase awareness among the people about disaster preparedness. Local knowledge and understanding in managing disasters or disaster preparedness’ is a concept that is highlighted in the trainings and formation of disaster management groups. Most of the beneficiaries’ are directly dependent on the rivers for transport or livelihood (fishing, shrimp collection). So they or their immediate family members have borne the brunt of several natural disasters in past and they have traditional knowledge on predicting and saving themselves during disasters.

For example, according to traditional knowledge sudden raise in humidity and calm wave-less rivers suggest an incoming storm surge. Sudden migration of fish schools also suggests upcoming storm. If the birds started chirping in the middle of the day and tried to flock in trees that also suggest an upcoming disaster according to traditional beliefs. Other important observations are that the water of the pond receded from the shores or the fishes act strangely during an advent of cyclones. Ants started to come out of the burrows and migrate inland, according to some it is also an indication of an upcoming storm. It is also true for tiger residing in the islands and crocodiles, which tried to migrate inland on the advent of cyclones. If a storm does impact during any voyage fisherman take their boats either inland before the storm hit and bind themselves with ropes on trees to deter being carried away by the rough waves. This information was cross-checked and found to be acceptable above 75 % amongst the group members and non-group members and so is considered as a consistent local knowledge on climate change adaptation strategies of the local vulnerable population. This knowledge, if communicated in a participatory way, can be invaluable for protection during disasters.

**Fig. 7:**
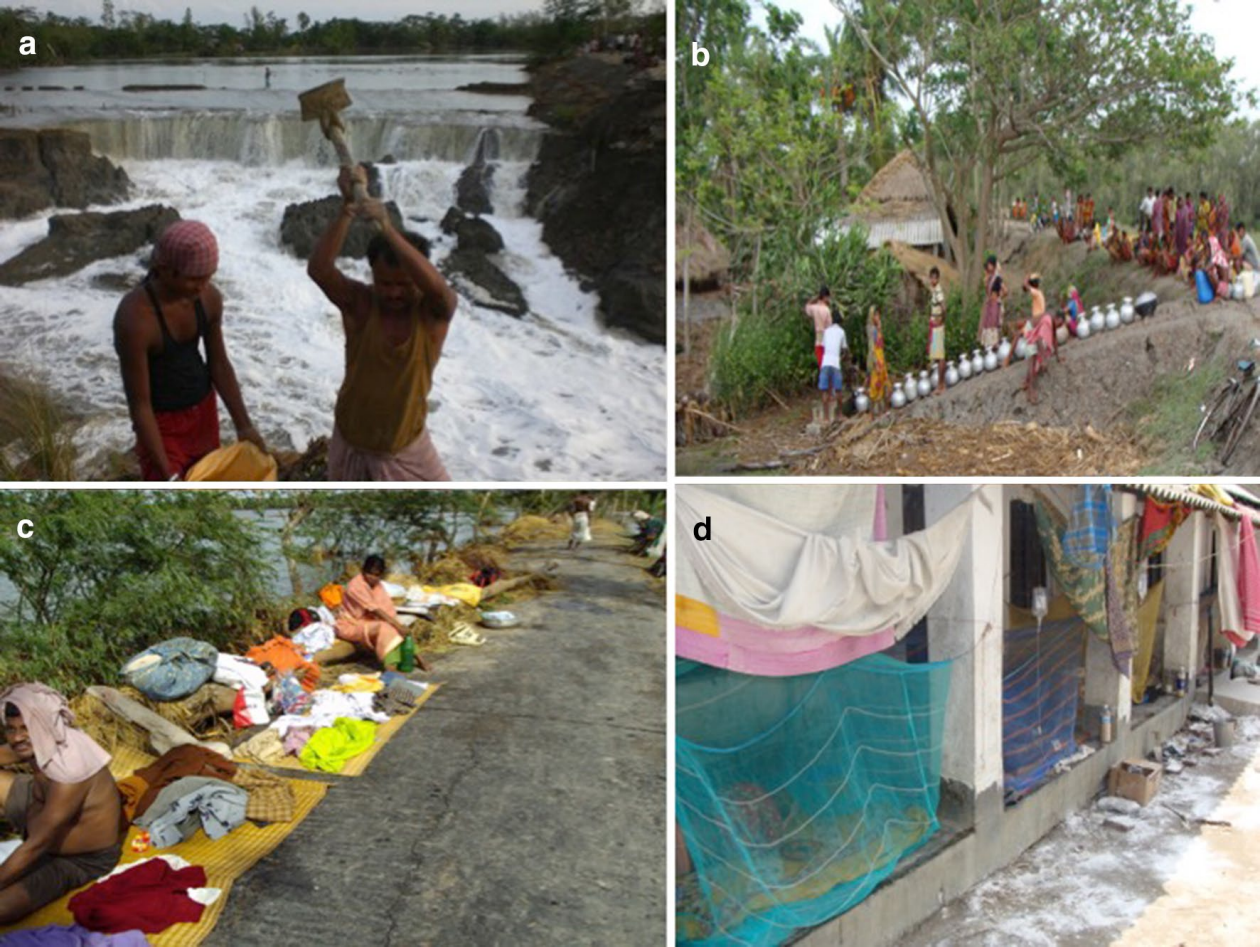
AILA devastations. **a** Sea water rushing in with the storm breaking the embankments and localities’ reconstructing the broken dams, **b** absence of fresh water-inhabitants have to wait with vessels for the water from relief to arrive, **c** displaced habitants whose houses have washed in flood water, **d** epidemic at the aftermath of the disaster and treatment at temporary shelters in absence of hospital facilities

The main problems during any natural disaster (Cyclone/Storm surges) is the breach of the earthen embankments along the riverside, hence the possibilities of salt water overflowing into the agricultural fields whole elevation is lower than the water level in most parts of the islands at Sundarbans (Fig. 7a). Immediately after any natural disaster, there arises an acute crisis of availability of fresh water, hence population has to depend on relief supply for their water demand, which in most cases are inadequate, poorly managed and unorganized as communicated by the trainees during interaction sessions (Fig. 7b). As the water rushes in during a cyclone by breaking the embankments, the huts near the shores are wiped out displacing the inhabitants (Fig. 7c). Lastly, the deaths of livestock animal/fresh water fishes due to drowning and purification of their flesh contaminate the fresh water sources resulting in epidemics like Cholera/Typhoid/dysentery and other water borne diseases amongst the stranded population. Governmental hospital facilities are inadequate, poorly managed, and under equipped to manage these emergencies. So NGO’s play a pivotal role is managing these health issues, but in the absence of space, temporary sheds are used to house the patients (Fig. 7d).

In these trainings and the disaster management team were taught to combat these issues, contact governmental/non-governmental stakeholders to manage the aftermath of any disaster. Basic medical/sanitation techniques were communicated to them like using boiled water for consumption during any waterlogged situation. The disaster management team was given all necessary knowledge to handle the emergencies and the effectiveness of these teams could only be tested during the situation of any disaster, but was a focal aim of this program to train a manforce that could be useful in the climate change community based adaptation strategy.

### Self-sufficiency and implementation of MFI

The attendance of group meetings by the beneficiaries’ was encouraging, over the phase of 2012–2013. The SHG and PCFC groups also managed to save substantial amount (2448.97 US Dollar by 2013) for emergency times (refer to Additional file 1: Table S3). Female groups (SHG) show more number of meetings than the PCFC because male members generally migrate to different reaches of the country to work as temporary labors during the summer months to augment their income during the lean (agriculturally unproductive) months unlike the women folks who stays back to look after the house and other landed property. Migration due to climatic variations is a common scenario worldwide. All the group members have saved substantial sums of money in their group accounts. It acts as a revolving fund as some members took lone from the same from the start of a new sustainable entrepreneurship or during any disaster. These savings also act as insurance from natural disasters and other calamities. Microcredit system was presented to the group members. Bank linkages were effective in introducing the notion of saving to the vulnerable communities. Non-governmental organizations (NGO) sometime effectively act as MFI provider to communities less accessible to the governmental support schemes (Premchander 2003). The successes of these schemes have invigorated the adaptation of this method of monetary sustainability to marginalized population that is not the part of the project group.

### Outcomes of PET

PET was organized at the end of the ‘Disaster management’ training programs which was the last component in the training schedules. Trainees, project workers were involved in acting and organizing the shows. This component was aimed to propagate the ideas of AIGA, MFI, Disaster preparedness’ to non group members, i.e. the whole community residing in and around Satjelia Island.

Theater troupes’ were taught to act an environmentally informative street drama amongst the trainees and mobile act shows were conducted in the market place, in local celebrations and even in other islands. At least 7 shows were coordinated in a month, where the notion of climate change, sea level rise, and resource conservation was highlighted and brought to the communities at large. There exists a fierce competition to be a part of this mobile theater troupes’ amongst the beneficiaries’ as they felt enthusiastic for fame and acknowledgement these bring to them. These non-economic benefits can also motivate community members to be a part of a conservation or a developmental scheme (Silva and Mosimane 2014). As more and more shows were conducted, it got an appreciable popularity amongst the marginalized community. And random verbal survey conducted within the spectators suggests interest and attachment to this program. This method also popularizes the group training endeavors and help to convey the message to the distant parts of Sundarbans whose inhabitants may have come to attend.

### Overall achievement of this project and future prospects

Participatory management processes have been a popular method to conserve natural resources (DasGupta and Shaw 2013; Chowdhury and Maiti 2014). Indian Sundarbans houses a dense human population along with it’s unique mangrove ecosystem. Industrialization and urbanization at the border of this mangrove habitat due the presence of a metropolitan Center like Kolkata has already been a hindrance to the proper conservation of the ecosystem as evident from recent studies (Chowdhury and Maiti 2016b). But the socioeconomic condition of the resident population makes it even more difficult to successfully implement any conservation activity without economically improving their status. In the absence of any proper employment opportunities’, low productivity of the agricultural lands due to the high salinity of soil/water compels the inhabitants to be dependent on resource exploitive income options like hunting for tiger prawn shrimps, poaching including illegal felling of mangrove trees for land conversion, overgrazing the mud flats by livestock etc.

This project introduced AIGA to the local population, spread awareness through technical as well as theoretical training sessions, preparing a skilled disaster management team that can serve the community during the frequent natural disasters like storm surges and cyclones, communicating the benefits of organic farming. Tropical/subtropical, aquatic as well as coastal ecosystems are exposed to escalating anthropogenic disturbances owing to ever-increasing human population, industrial development and land conversion activities (Maiti and Chowdhury 2013; Chowdhury and Maiti 2016a, b). Recent research works have shed light into the fact that in a fragile amphibious ecosystem like Sundarbans, even the conservation friendly practice like ecotourism can have a deleterious effect on the ecosystem, contributing to the Pb pollution due to seepage of low grade fossil fuel into conserved waters from tourist/ferry boats (Chowdhury and Maiti 2016a, c). Pollution can have a multitide of direct and indirect effects that can distabilize a ecosystem by the processes of biomagnification, bioaccumulation in flora and fauna along with degredation of the abiotic environment (Kumar and Maiti 2015; Kumar et al. 2015; Banerjee et al. 2016; Naz et al. 2016; Chowdhury and Maiti 2016a, b, c). So identification and introduction of resource friendly AIGA are essential before planning any conservation effort in this region. Sustainable, environmentally friendly alternate income generation activities like Poultry, small business like grocery shop, Tricycle van, etc. can augment the economic condition of the local population. Participatory mode of conservation through NGO’s and community based organizations (CBO’s) are experimented in different parts of the globe with successful outcomes (DasGupta and Shaw 2013; Chowdhury et al. 2016). The success of the project components (AIGA, Plantation program, Sustainable agriculture, Disaster management committee and MFI) was monitored by the project employees (14 field supervisors, 1 Livelihood support officer, 1 Greening officer) on the instruction of the ‘Program Coordinator’ and the project progress has again been periodically monitored by the inspection team of the funding agency. The beneficiaries’ selected for a particular support (AIGA) by the group organization was given technical help even after they have engaged into the trade. Information about governmental subsidies on poultry medicines were communicated to them so that they could self sustain their business and avail the maximum help from the governmental and non-governmental institutions. Again the beneficiaries’ recorded the progress of the business and that was used for understanding the success of their trade. In case of problems, experts immediately assist them in coordination of the project employees to identify the problem and to solve the issue.

Loss of traditional knowledge was also observed in other tropical communities affected by natural disasters like typhoon (Acosta et al. 2016). And any natural disaster leads to a panic situation that reduces the ability of the community to deal with the immediate emergency (Asuero et al. 2012). Ten disaster management committees would serve a pivotal role in disaster preparedness’ and management during any natural calamity. Local knowledge on adaptation to natural disaster has also been taken into account so that the same can aid in emergency situations and not get lost with time and development.

Chemical pesticides and fertilizers have a deleterious effect on the agricultural lands with limited fertility as in case of Indian Sundarbans. Introducing organic farming methods had been an arduous task amongst the farmers, already dependent on the chemical fertilizers for generations. So 1015 beneficiaries were selected as per the recommendation of the group, who were given support/technical trainings to practice organic farming on a part of their land. This act as success stories to the community at large to get interested in practicing the organic agriculture.

Plantation program aims to vegetate the naked mudflats by community participation. One of the focal reasons for mangrove deforestation in the human habituated islands of Sundarbans had been overgrazed by livestock specially goats apart from land conversion activities. So involving the community to plant the saplings gave them an emotional attachment with the planted saplings and it reduces their overgrazing activity. This conclusion can be drawn from the survival rate of the saplings and informal interviews conducted by the project employees amongst the non-group members of the community residing near the plantation area.

PET helped to convey the messages of this multi-pronged climate change mitigation endeavor to different parts of the delta in the form of easy to understand the plays with participatory involvement of the audiences. The success of this communication method would be revealed in the future if other non-group members also adopt these processes.

Similar conservation methods can be employed in other areas of the globe facing climate change problems and harboring vulnerable population. The tropical rainforests/mangrove patches/glacial wastelands/temperate grasslands in South East Asia, African forests, Brazil, Andean hot spot, island atolls of Micronesia/Polynesia and artic settlements of Canada are to mention a few of the regions that are susceptible to climate change related disaster issues as well as anthropogenic disturbances and are focusing on participatory climate change adaptation strategies as a sustainable solution (Berkes and Jolly 2002; Mirza 2003; Morton 2007; Engle and Lemos 2010; Gero et al. 2010; Duru et al. 2012). And common root cause hindering any conservation activity in these diverse geographical locations with different socio-economic complexities is the unstable economic condition of the resident population. So ‘lecturing’ local people about climate change ‘problems’ from a scientific perspective can never be a effective practical solution in these vulnerable areas. AIGA initiatives should be included in planning any climate change mitigation strategy to deal with this economic disparity before venturing into implementation of any conservation plans. Community should be motivated to conserve their habitat out of their own ‘free will’ and not due to ‘governmental directives’ which can only be achieved by ensuring their active participation in any environmental management plans.

## Conclusions

This paper shed light into a group-based awareness, training campaigns’ to communicate the affect of climate change induced disaster to the vulnerable, marginalized population of the last human habitable island of Indian Sundarbans (Satjelia). Populations were organized into group-based structure (2100 families) through the help of wealth rank tool and PRA. Training on various aspects of management of climate-induced problems was conducted like sustainable agriculture, alternative livelihood options, disaster mitigation and resource conservation. They felt their contribution to this program when their time devoted for attending these training or working for protection of the mangroves are converted into their local contribution according to the prevalent labor rates for this socio-environmental program. participatory educational theater (PET) was also introduced with success to spread the idea of these activities and climate change related awareness amongst the population of sundarbans at large. Integrating traditional knowledge with modern disaster predicting tools disaster management groups are made and a section of society is created who could protect themselves and their community members during any crisis. Trainees successfully implemented the poultry farming, sustainable agriculture and taken active role in plantation and conservation of mangroves which is a success of this endeavor. 87.07 ha of naked mudflats were brought under mangrove cover due to the participatory engagement of the group members which is a direct step to combat climate change by trapping its principal contributor the carbon-di-oxide through plantation of mangroves. And this project aims to bridge this gap between scientific understanding of climate change and practical ‘problems’ at the disaster torn, poverty affected, marginalized community at Indian Sundarbans. And this multi-pronged adaptation approach can be effectively and practically used in other similar locations around the globe plagued by climate change issues and looking for a sustainable adaptive strategy.

## Additional file

10.1186/s40064-016-2816-y Sociological complexities in Indian sundarbans, the structure of the project and MFI savings.
