# Supplementary material for: How to communicate climate change ‘impact and solutions’ to vulnerable population of Indian Sundarbans? From theory to practice
Source: Springerplus. 2016 Jul 29;5(1):1219. doi: 10.1186/s40064-016-2816-y (PMC4967050; doi:10.1186/s40064-016-2816-y)
Supplement: Supplementary file 1 — 10.1186/s40064-016-2816-y Sociological complexities in Indian sundarbans, the structure of the project and MFI savings. [file 40064_2016_2816_MOESM1_ESM.docx]

**Additional file 1**

**How to communicate climate change ‘impact and solutions’ to vulnerable population of Indian sundarbans? From theory to practice**

By,

Abhiroop Chowdhury^1*^, Subodh Kumar Maiti^2^ and Santanu Bhattacharyya^3^

^1^ Research Scholar, ^2^Professor, Department of Environmental Science and Engineering, Indian School of Mines, Dhanbad- 826004, India. ^1*^Email: [abhiroop.chowdhury@gmail.com](mailto:abhiroop.chowdhury@gmail.com), ^4^[skmism1960@gmail.com](mailto:skmism1960@gmail.com). * Corresponding Author.

^3^Programme Coordinator, Tagore Society for Rural Development, 46B Arabinda Sarani, Kolkata- 700005, India. Email: [santanu.bhattacharyya1@gmail.com](mailto:santanu.bhattacharyya1@gmail.com)

**Sociological complexities in Indian sundarbans, the structure of the project and MFI savings**

1. Social considerations in Indian sundarbans

The present human settlement in Sundarbans is largely a result of human migration that started during British Raj, by Claude Russel during 1770-1773 and is still continuing. The main motivation is to convert the mangrove lands into productive agricultural tracts, with total disregard for mangrove conservation. The settlers have come from distant reaches of India (Chotanagpur, Hazaribag, Manbhum of present day Jharkhand state, Balasore in Odissa state; Birbhum, Bankura, Midnapore, Nadia of West Bengal State; Jessore of Bangladesh and even from Arakan region of Myanmar (Jalais, 2010). Present population of Indian sundarbans is a mixture of religion, caste and tribes. Indian society is unique for it’s division in basis of caste, class, region, religion and sex (Chanana, 1993). These austere regions have a huge proportion of the reserve castes, which have a lower standing in Indian society than their other counterparts (table 1).

Table 1: Population and ethnic composition of Satjelia island (collected from census data 2001).

| **Sl. No** | **Name of the Hamlet** | **Population** | **Scheduled Caste (%)** | **Scheduled Tribe (%)** | **Illiterate (%)** |
| --- | --- | --- | --- | --- | --- |
| **1** | **Sudhanshupur** | 4548 | 63.2 | 36.8 | 39.14 |
| **2** | **Hamiltonabad** | 3515 | 74.17 | 25.83 | 42.14 |
| **3** | **Satjalia** | 8243 | 76.1 | 6.8 | 38 |
| **4** | **Dayapur** | 4444 | 83.8 | 14.2 | 32 |
| **5** | **Sadhupur** | 6387 | 85.2 | 10.9 | 40 |
| **6** | **Lahiripur** | 6637 | 96.3 | 0.0 | 34 |
| **7** | **Luxbagan** | 4263 | 76.1 | 12.9 | 31 |

In Indian pretext caste is arranged in a tier system with top tiers of Brahmins (priestly caste), followed by Khatriya (warrior/ruling caste) getting the most social standing, then comes Vaisyas (Businessman caste) and the so-called polluted/untouchable ‘Sudra’ are at the lowest tier of the social order. According to Chanana, 1993, 82% of Hindu population in India comprises of this lower ostracized caste. Caste or ‘jati’ can be defined as an endogamous, hereditary, hierarchically arranged group. These far reaches of the country is mostly populated by these lowest strata of the caste who technically falls under the ‘protective discrimination’ category of Scheduled Caste (SC) and Scheduled tribes (ST) and socially as ‘Sudra’, who by governmental systems and legal legislations are given numerous socio-economic benefits to close this caste divide and augment their economic standing in the society (Xaxa, 2001; Chanana, 1993). These local habitants are a floating population without any sense of home coming from different reaches of the nation, ostracized by the high caste dominated Indian Hindu society and still migrating as seasonal labors to far off places for employment in absence of proper livelihood and less-productive land in this place. Muslim population is also in this region that have migrated from Islamic nation of Bangladesh through the less protected, forested, porous border between the two nations through the myriads of crisscrossing river networks of the delta. They are regarded as minority in the Indian society sharing almost similar discrimination as SC/ST’s, and treated as polluted class (*Mlecha*) by the high caste (Brahmin and Khatriya) dominated society.

2. Project Initiation and formulation

Social livelihood programs are widely used for improve the economic condition of the marginalized populations in these vulnerable regions. Satjelia Island (Fig 1-Manuscript) is the last habitable island of Indian Sundarbans communicable only by waterways, with sporadic electricity crisis and majority of population falling under category of Bellow Poverty Level (BPL) and belonging to ostracized minority population (SC/ST, Muslim).

The problem of environmental degradation is addressed in the project through understanding and linking livelihood, trainings and vulnerability. The problem and solution is explained in the figure 1.

The problems of deforestation, land conversion leads to global warming and that in turn leads to melting of ice caps and sea level raise which again increase the frequencies of natural disasters destabilizing this costal ecosystem. Solution is envisioned to be spreading awareness about the negative impacts of the climate change, changing human perspective through training programs and plantation program of mangroves by involvement of local population. This project is funded by BMZ (The Federal Ministry for Economic Cooperation and Development, Germany), KKS (Karl Kübel Stiftung für Kind und Familie, Germany) and implemented through the local non-governmental organization (NGO) of Tagore society for Rural Development, India (TSRD).

Local contribution (LC) has been allocated to be 6.9% of the total budget which as per agreement, that could be valuated or contributed by the beneficiaries’ or implementing local NGO, i.e TSRD. Total expenditure up to 2012 to 2015 is 81.9% of the total budget amount (FC+LC) and total LC collection from 2012 to 2015 has been10.23% of the total expenditure. Viewing success of the project components and responses’ from the target population, it has been extended till December, 2016 by the mutual accord of the donor and implementing NGO.


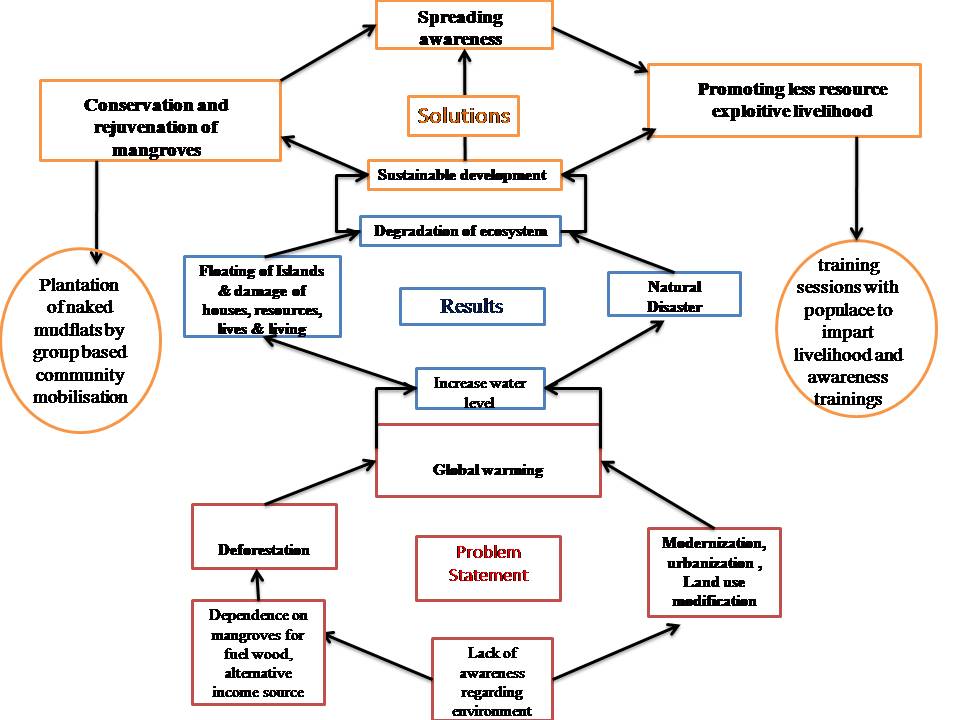


Figure 1: The problem, solution tree on which the project is designed

Table 2: Group organization and hierarchy

| **Committee** | **Number** | **Each unit participation** | **Members** |
| --- | --- | --- | --- |
| Apex Committee (PCFC+SHG) | 1 | 6 | 6 |
| Village Committee (PCFC+SHG) | 1+1 | 36 | 72 |
| PCFC+SHG | 1050+ 1050 | 15 | 2100 |

Table 3: Total savings of SHG and PCFC group as per village hamlet

| **Sl.No** | **Name of Revenue Village** | **Total Savings of SHG (INR)** | **Total Savings PCFC (INR)** | **Total Savings of Revenue Village (INR)** | **Total Savings of Revenue Village (US Dollar)** |
| --- | --- | --- | --- | --- | --- |
| 1 | Dayapur | 57,000.00 | 59,132.00 | 1,16,132.00 | 1,733.31 |
| 2 | Satjelia | 56,677.00 | 62,970.00 | 1,19,647.00 | 1,785.78 |
| 3 | Sudhangshupur | 84,000.00 | 45,840.00 | 1,29,840.00 | 1,937.91 |
| 4 | Luxbagan | 69,589.00 | 65,262.00 | 1,34,851.00 | 2,012.70 |
| 5 | Lahiripur | 1,53,449.00 | 1,57,239.00 | 3,10,688.00 | 4,637.13 |
| 6 | Sadhupur | 56,040.00 | 63,236.00 | 1,19,276.00 | 1,780.24 |
| 7 | Hamiltonabad | 54,000.00 | 66,000.00 | 1,20,000.00 | 1,791.04 |
|  | Total | 5,30,755.00 | 5,19,679.00 | 10,50,434.00 | 15,678.12 |

**Reference**

Chanana, K. (1993). Accessing higher education: the dilemma of schooling women, minorities, Scheduled Castes and Scheduled Tribes in contemporary India. Higher Education 26(1): 69-92.

Jalais, A. (2010). Forest of Tigers People, Politics and Environment in the Sundarbans. Routledge, New Delhi.

Xaxa, V. (2001). Protective discrimination: why scheduled tribes lag behind scheduled castes. Economic and Political weekly 36(29): 2765-2
